# Affect Modulated Startle Response in Anorexia Nervosa, Restricting Type: Implications for Theory and Practice

**DOI:** 10.7759/cureus.27304

**Published:** 2022-07-26

**Authors:** Fauzia Mahr, Scott C Bunce, Roger E Meyer, Katherine A Halmi

**Affiliations:** 1 Psychiatry and Behavioral Health and Pediatrics, Penn State College of Medicine, Hershey, USA; 2 Psychiatry and Behavioral Health, Penn State College of Medicine, Hershey, USA; 3 Psychiatry, Weill Cornell Medical Center, Ithaca, USA

**Keywords:** depression, anxiety, emotional categorization, affect modulated startle response, anorexia nervosa

## Abstract

Objective: Individuals with anorexia nervosa (AN), restricting type demonstrate unique emotional responses to hedonically positive stimuli beyond eating disorder (ED)-related stimuli. The goal of this study was to evaluate differences in responses to five types of emotionally positive stimuli among acutely ill anorexia nervosa (IAN), restricting type patients, weight-recovered anorexia patients (WRAN), and healthy controls (HCs) using affect modulated startle response (AMSR) as an objective measure.

Method: A total of 28 participants were recruited (n=28). Fourteen participants were recruited as IAN using the Diagnostic and Statistical Manual of Mental Disorders-V (DSM-V) criteria, seven were WRAN, and seven were HC females. All participants were female and aged between 8 and 18 years. The participants viewed images depicting negative, neutral, standardized, and non-eating disorder (ED)-related positive stimuli. Additionally, four categories of ED-related stimuli (high-calorie food, body image, success, and parent-child relationships) were presented to all participants during a standard AMSR paradigm.

Results: No significant between-group differences were found for any of the four ED stimulus categories; all groups showed an inhibited startle response to the four ED-related categories. In contrast, IAN and WRAN showed reduced hedonic responses to standardized positive stimuli relative to HC-replicating previous results. Reduced hedonic response to the standardized (non-ED) positive stimuli was highly correlated with self-reported social anxiety, low self-esteem, body dissatisfaction, asceticism, interpersonal problems, and ineffectiveness.

Conclusion: AN patients had a reduced hedonic response to some non-ED-related positive stimuli, which correlated with several anxiety-related traits. In contrast, their early automatic responses to high-calorie food, normal weight models, images of success, and positive parent-child relationships did not differ from HC, suggesting these stimuli are either being evaluated as highly interesting or hedonically positive.

## Introduction

Anorexia nervosa (AN) is a debilitating psychiatric disorder characterized by an intense fear of weight gain, body image distortions, self-starvation, and dysphoric mood. Individuals with restricting type anorexia nervosa (AN-R) are often highly anxious, ascetic, and anhedonic, reporting few pleasures in life beyond the pursuit of weight loss, characteristics that often persist following weight recovery [1]. Current acute treatment of AN relies on behavioral interventions designed to alter misperceptions of body image, responses to food, and perceptions of hunger and satiety. However, the high rate of relapse suggests that current treatment strategies may not be addressing important aspects of the underlying pathology. Although much is still unknown about the neuropathophysiology underlying AN-R, growing evidence indicates that altered brain reward systems and aberrations in emotion regulation (hedonic processing) that go beyond disorder-specific stimuli (i.e., food and body image) play an important role in the development and maintenance of AN. A better understanding of the disorder-specific differences in the processing of appetitive and aversive stimuli among AN-R would help clinicians develop more informed and targeted treatment strategies.

A recent study by Friederich et al. [2] used affect modulated startle response (AMSR) to evaluate the motivational significance of both eating disorder (ED)-specific and standard (non-ED) emotional cues in AN-R and bulimia nervosa (BN). AMSR, a widely used psychophysiological metric of motivational state (i.e., approach or withdrawal) and emotion-related attention, was employed because it is also largely independent of conscious, intentional control. The AMSR metric is based on the consistent finding that the emotional state of an organism reliably influences the magnitude of the eyeblink startle reflex. AMSR provides a broad index of an individual’s current motivational state. These early stages of emotional evaluation do not rely on conscious, or “controlled,” evaluative processes. Rather, these early, “automatic” processes (<200 ms after stimulus onset) occur prior to conscious awareness and play an important role in shaping more complex emotions, attitudes, and behaviors that do rely on conscious experience.

Friederich et al. [2] found that AN-R did not show startle inhibition, which is an appetitive/approach response to images depicting palatable food, body images, or generalized, non-ED-specific positive images. Due to their general lack of startle inhibition across these stimulus categories, Friederich et al. [2] suggested that AN-R showed a generalized failure to activate the appetitive motivational system. Importantly, though, control participants did show the expected startle inhibition pattern to the non-ED positive images, but they did not show the expected inhibition to the food images, and the responses of healthy controls (HCs) and AN-R to food images did not differ. Given that healthy controls, unless fully satiated, tend to show startle inhibition to palatable food cues [3], this finding suggests the hypothesis regarding response to food (and possibly body image) was not adequately tested.

Recent neuroimaging research suggests that the brain reward circuits (anteroventral striatum, insula, and prefrontal cortex) of AN-R women may be more responsive to ED-related stimuli than healthy controls, but less responsive to non-ED-related rewards [1]. A functional magnetic resonance imaging (fMRI) study of neural responses during a simple guessing game with monetary rewards in AN suggested that weight-recovered AN-R (WRAN) women may utilize cognitive control strategies to compensate for less precision in their capacity to modulate affective responses to stimuli indicating losing versus winning money. In addition, this study found that questionnaire measures of trait anxiety among the AN-R correlated with the magnitude of the brain response during both wins and losses. Thus, the most anxious women with AN-R responded to the emotional stimuli in an overly “cognitive” manner, making it difficult for them to respond to the rewarding outcomes of their choices.

Neurotransmitter changes in response to anxiety-provoking stimuli can lead to attentional biases towards a feared stimulus. Executive function abnormalities seen in ED patients increase the likelihood of attending to feared stimuli. Activity in the left prefrontal cortex influences this visual attention [4].

Moreover, anticipatory anxiety can lead to stimulus avoidance and the exaggerated anxiety response to food cues in AN patients may alter the valuation of food-related stimuli. “Alterations in the valuation of rewarding and aversive stimuli" may play a key role in AN development and maintenance [5].

The purpose of this pilot study was to use an objective metric of emotional/motivational categorization, the AMSR, to evaluate the responses of AN-R to five distinct categories of putatively positive (naturally rewarding) ED-related categories; high-calorie food, body image, success and positive parent-child relationships, and non-ED-related stimuli, in addition to emotionally neutral and negative stimuli among ill-anorexia nervosa (IAN), WRAN, and HC. Self-reported measures of anxiety-based traits (social anxiety, low self-esteem, rigidity, over-control, and asceticism) were used to evaluate the relationships between trait anxiety and AMSR.

## Materials and methods

Participants

Twenty-eight adolescent females, ages between 8 and 18 years, were recruited for the study. This study was approved by the institutional review board at the Penn State College of Medicine (Approval #37745). All participants signed informed assent/consent forms. Inclusion criteria for AN included a diagnosis of AN-restricting type based on a psychiatric interview using the Diagnostic and Statistical Manual of Mental Disorders-V (DSM-V) criteria and admission to the eating disorders partial hospitalization program at Children’s Hospital at Penn State Hershey Medical Center. Psychiatric interviews were administered by clinicians with expertise in eating disorders (FM, ML, and RL). To be considered “weight recovered,” participants: (1) previously met the criteria for AN under DSM-V, (2) had maintained more than 90% of ideal body weight (IBW) for a minimum of three to six months, and (3) had not binged, purged, or engaged in restrictive eating patterns for three to six months. Exclusion criteria include psychotic illness, suicidal ideation, substance abuse/dependence, or post-traumatic stress disorder (PTSD). HC adolescents with no history of psychiatric, neurological, or severe medical disorders were recruited through referrals from local family physicians. Participants completing the study were: WRAN, IAN, and HC.

Self-report instruments included the Children’s Depression Inventory (CDI), Eating Disorders Inventory-37, and the Revised Children’s Manifest Anxiety Scale [6]. One IAN participant opted not to complete the AMSR study and one HC participant’s data were confounded by excessive artifacts.

Affect modulated startle response

The eyeblink is part of the startle reflex, a defensive response that serves to protect the body from harm following a perceived threat and can be reliably elicited using an abrupt acoustic stimulus. In a negative or withdrawal state (fear, disgust), the magnitude of the eyeblink is amplified relative to a neutral state. If the organism is in a positive or approach-related state (appetitive, interested), the magnitude of the eyeblink is decreased relative to a neutral state. To the degree that they influence the motivational state of the organism, positive and negative stimuli affect the magnitude of the startle response to acoustic stimuli [4]. 

Stimuli

Non-ED stimuli consisted of negative, neutral, and positive images are drawn from the International Affective Picture System. Positive stimuli were chosen by consensus among three ED experts to be experienced positively by young anorectic females (FM, ML, and RL), and consisted of images depicting cute animals, pretty gardens, etc. Images of high-calorie food, body image, “relationships,” and “success” were drawn from the internet. Body image stimuli depicted young, normal-weight models in attractive, but not revealing, clothing (e.g., sundresses with bare arms). Relationship images depicted positive adult-child interactions. Success images depicted academic success (A+ on papers), gold cups and blue ribbons, and athletic success.

Procedure

Participants viewed 84 color images for six seconds each, with an inter-trial interval (ITI) varying from 16 to 24 seconds. An acoustic startle probe (50 ms, 105 dB white noise, and instantaneous rise time) was presented during the slide-viewing period on 63 trials; 12 startle probes were also presented during ITI (blank gray screens). AMSR was calculated as the mean amplitude of trials for each category.

Startle probes were presented binaurally through headphones with the presentation and timing of stimuli controlled by Neuroscan's stimulus presentation application (Neuroscan, Inc., Charlotte, NC, USA). Eye blinks were assessed by recording electromyographic (EMG) activity from 4 mm Beckman Ag/AgCl electrodes positioned over the orbicularis oculi muscle beneath the left eye using Neuroscan’s SynAmps Bioamplifier and SCAN software (Compumedics, Charlotte, NC, USA). The EMG channel was digitized continuously at 1000 Hz (bandpass filter, 90-1000 Hz; time constant, 125 ms) [7]. Startle data were epoched offline from 50 ms prior to probe onset until 150 ms after probe onset. Blinks were scored for magnitude and visually inspected to control for artifacts. Responses were defined as changes from the mean EMG level of the 50 ms prestimulus baseline period to peak amplitude between 20 and 150 ms after probe onset. Trials with artifacts or excessive noise in the baseline period were eliminated.

Statistical analyses 

Based on the Friederich et al. [2] findings, a 2 × 2 mixed-model analysis of variance (ANOVA) was used to test a priori contrasts between IAN and HC responses to neutral and positive stimuli. A priori hypotheses for the four categories of ED-relevant stimuli were tested using 3 × 2 mixed-model ANOVAs, with a group (IAN, WRAN, and HC) as a between-subject factor and stimulus type (neutral versus ED-relevant) as a within-subject factor. Post-hoc analyses consisted of paired t-tests for within-subject differences. Statistical analyses were computed using SPSS version 21.0 (International Business Machine (IBM) Inc., New York, USA) [8].

## Results

Demographic data are presented in Table 1.

**Table 1:** Demographic characteristics of underweight anorexic, weight-recovered anorexic, and healthy control participants. (A) F-value or c^2^ (serotonergic meds) for group differences in demographic characteristics; ANOVA degrees of freedom = (2, 23); c^2^ degrees of freedom = 2. (B) Significant post-hoc comparisons, Tukey's honestly significant difference test (Tukey’s HSD) for (a) AN versus WRAN, (b) AN versus controls, and (c) WRAN versus controls. p < 0.05; *p < 0.01; ^†^p < 0.001. BMI: body mass index; CDI: Children’s Depression Inventory; AN: anorexia nervosa (DSM-IV); serotonergic meds: percentage of the group on serotonergic medications.

	IAN	WRAN	Controls	c^2 ^or^ A^		
Measure	Mean (SD)	Mean (SD)	Mean (SD)	F-value	p	Post-hoc^B^
Age	13.46 (1.66)	16.00 (0.89)	14.14 (1.35)	6.34	0.006	a*, c
BMI	15.19 (1.74)	19.27 (1.68)	20.01 (2.58)	16.85	0.000	a^†^, b^†^
CDI T-score	58.54 (12.03)	49.00 (14.63)	40.86 (6.20)	5.58	0.011	b*
Serotonergic meds	23.1%	100%	0%	15.80	0.000	a*, c*

One-way ANOVAs followed by post-hoc analyses revealed that (1) WRAN was older than IAN and had higher BMI scores; (2) in other respects, the subjects did not differ from each other; and (3) IAN had higher CDI scores than HC.

**Figure 1:**
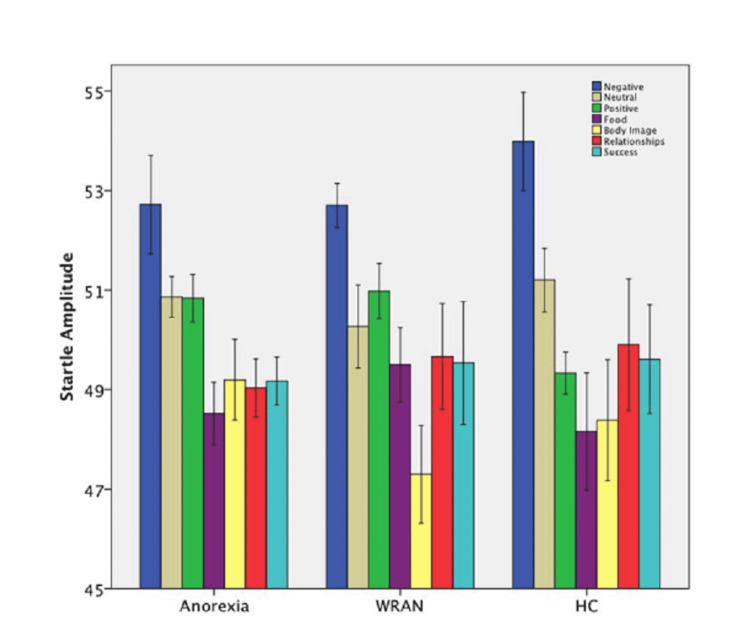
Affect modulated acoustic startle response amplitude in ill anorexia nervosa (IAN), weight-recovered anorexia nervosa (WRAN), and healthy control (HC) participants. Error bars = +1 standard error. Startle amplitudes represent T-scores. Positive (green bars) represent responses to non-eating disorder-related positively valenced stimuli (e.g., puppies and kittens).

The planned neutral-positive stimulus contrast between IAN and HC revealed a main effect for stimulus (F (1, 18) = 4.67, p = 0.045) that was qualified by a group × stimulus interaction (F (1, 18) = 4.39, p = 0.05). The planned neutral-positive stimulus contrast between IAN and HC revealed a main effect for stimulus (F (1, 18) = 4.67, p = 0.045) that was qualified by a group × stimulus interaction (F (1, 18) = 4.39, p = 0.05). Inspection of the means revealed that, whereas HC showed the expected startle suppression to positive stimuli, IAN startle amplitudes were not suppressed in response to the positive stimuli (see Figure 1).

**Figure 2:**
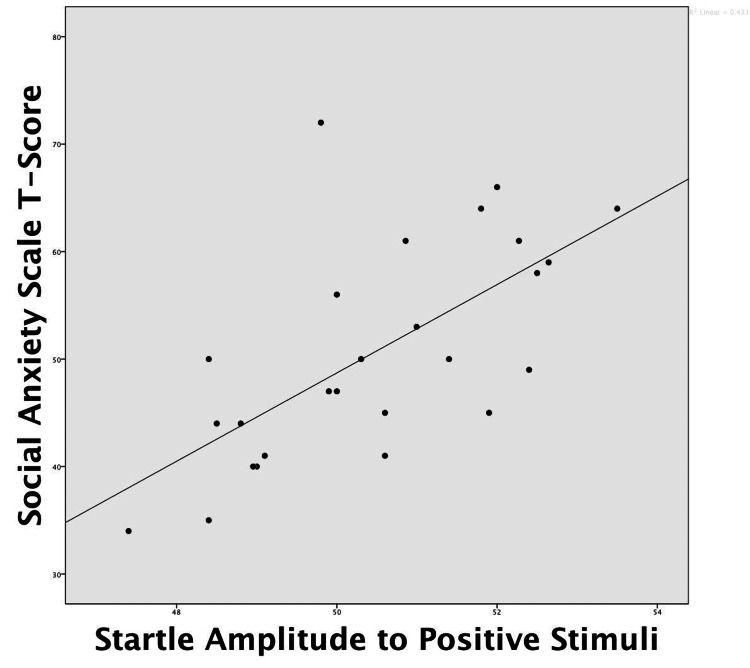
Affect modulated acoustic startle response amplitude and correlation with social anxiety. Social anxiety = T-score on the revised children’s manifest anxiety scale.

Like the IAN, WRAN did not show suppressed startle in response to the positive stimuli relative to HC (F (1, 11) = 2.41, p = 0.035). The results for the HC and the two patient groups in response to ED-related stimuli showed a similar pattern across the four stimulus categories, i.e., relative to the neutral stimuli, the startle response was attenuated in response to the ED-related stimuli for food (F (1, 23) = 9.35, p = 0.006); body image (F (1, 23) = 12.28, p = 0.002); and success (F (1, 23) = 4.28, p = 0.05); with a similar trend for relationships (F (1, 23) = 3.07, p = 0.09). There were no interactions within groups (all F’s < 1.0). Whereas all three groups appeared to have attenuated responses to the ED-related stimuli (F’s < 1.0), within-group paired student’s t-tests revealed that AN showed active suppression of ED-related stimuli relative to neutral stimuli for each of the four categories (see Figure 1; food, t (1, 12) = 2.63, p = 0.022; body image, t (1, 12) = 2.18, p = 0.050; relationships, t (1, 12) = 2.78, p = 0.017; success, t (1, 12) = 2.33, p = 0.038). The amplitude of the AMSR to positive stimuli was correlated with social anxiety (Pearson’s r = 0.66, p < 0.001; see Figure 2), the CDI (r = 0.39; p < 0.05), as well as 10 Eating Disorder Inventory (EDI-3) scales, ranging from personal alienation (r = 0.41, p = 0.03), through interpersonal alienation, overcontrol, interpersonal insecurity, drive for thinness, asceticism, interpersonal problems, body dissatisfaction, and ineffectiveness, to low self-esteem (r = 0.59, p = 0.001). 

## Discussion

The purpose of this pilot study was to evaluate the hypotheses that (1) patients with anorexia nervosa, restricting type show evidence of aberrant emotional processing in response to putatively positive affective stimuli; and (2) that differential emotional processing of these images might be linked to known traits in AN-R. Compared with HC, both IAN and WRAN failed to demonstrate startle suppression (positive hedonic responses) to typically positive stimuli, a finding that is consistent with findings reported by Friederich et al. [2]. Failure to respond to the positive stimuli in the startle paradigm correlated with personality characteristics commonly found in AN-R patients, i.e., less hedonic response was associated with greater social anxiety, lower self-esteem and greater body image distortions, asceticism, interpersonal problems, and a sense of ineffectiveness. ED and AN-R, in particular, have been associated with anxiety and anxious-avoidant, perfectionistic, and obsessive-compulsive traits, which persist even after recovery and have an increased prevalence among unaffected family members [9-12].^ ^Anxiety disorders often precede the onset of AN-R [13,14] and social phobia, anxiety disorders, and obsessive-compulsive disorders are common comorbidities in AN-R. Indeed, Strober et al. [15] suggested that AN could be conceptualized as a form of anxiety disorder with a proclivity towards rapid fear learning and high resistance to extinction.

In contrast, our results indicated that IAN and WRAN did not differ from HC in their reduced startle responses (positive hedonic responses) to ED-related stimuli, including high-calorie foods, attractive normal-weight body images, and images of success, with a similar trend in response to images depicting positive emotional relationships between parents and children. The finding that IAN and WRAN showed startle suppression to high-calorie foods as well as normal-weight body images, suggests an early, positive hedonic response to or intense interest in ED-related stimuli. Whereas this may seem inconsistent with the typical self-reported aversion to food and normal-weight body images, it is consistent with the finding that AN-R shows enhanced levels of attention, or attentional biases, to ED-related stimuli [4,16]. The startle response is modulated by attention as well as the valence of the participant’s reaction to a stimulus, and under some circumstances, attentional processes can override the influence of valence on the startle amplitude [17].

Our results regarding the failure to respond to some non-ED-related positive stimuli in anxious anorectic patients replicate some of the findings from the literature regarding impairment in the processing of emotionally relevant stimuli [2,14]. Heightened attention towards body images and weight-related stimuli, and away from positive stimuli, has been reported in patients struggling with eating disorders [4]. According to Aspen et al. [4], the excessive anxiety seen in AN patients may be one of the factors leading to heightened attention to feared stimuli such as high-calorie food and body images.

As an alternative hypothesis to increased attention, Kaye et al. [18] proposed that differences in hedonic responses to ED-related stimuli may occur later in the evaluative process, when more cognitive control mechanisms come into play. These authors argue that the neurocircuits involved in the initial identification of the hedonic significance of a stimulus include the amygdala, ventral striatum, insula, and ventral areas of the anterior cingulated cortex and prefrontal cortex (“ventral circuits”). A dorsal neurocircuit, comprised of the hippocampus, dorsolateral prefrontal cortex (DLPFC), dorsal regions of the caudate, parietal cortex, and other regions, is thought to modulate later executive and cognitive control functions. Kaye and colleagues [18] hypothesize that individuals with AN have exceptional self-control and an ability to inhibit appetite as a function of the strength of this dorsal cognitive circuit. Excessive utilization of cognitive strategies may play a critical role in the ability of patients with anorexia to restrict oral intake despite the presence of an initially positive automatic response to food. Our data are consistent with this theoretical model. However, in our paradigm, we could not determine if an attentional mechanism accounts for the suppressed startle; or, whether the positive emotional salience related to ED-related images is suppressed by consequent cognitive processes. We plan further studies to tease these issues apart. In this work, it will be important to determine if similar results would be observed using the primary reinforcing stimulus (e.g., taste) as compared with the visual food-related stimuli.

From a clinical perspective, the relatively strong relationship between self-reported anxiety and reduced hedonic response to non-ED rewards suggests the potential that reduction of anxiety might help restore a more positive hedonic response to non-ED stimuli. Some authors have suggested that anxiety-based treatments such as attention bias modification (ABM) might increase success rates in ED treatment [3,19]. ABM attempts to identify the patient’s specific biases and then employs specified tasks to train the patient’s brain to abort the predominant attention to those cues. The existing research indicates that ABM plays a key role in modifying cognitive processes in the prefrontal cortex rather than the anxiety experienced in earlier phases of response to stimuli [4]. This approach to the treatment of patients with anorexia nervosa would be consistent with the model proposed by Kaye and colleagues [18], which highlights the importance of cognitive circuits in modifying the evaluation of emotional stimuli among these individuals.

Finally, the results of the present study need to be compared with the results reported by Friederich et al. [2]. These investigators did not find positive hedonic responses (startle suppression) to ED-related cues. Importantly, they also failed to find startle suppression to these cues among healthy controls. In essence, neither in the present study nor in the work of Frederich and colleagues [2], did IAN nor WRAN differ from HC in their responses to the ED-related stimuli. Nevertheless, it remains an open question as to why the current study demonstrated positive hedonic responses to ED-related stimuli among anorectic patients and control participants when the Friederich et al. [2] study did not. Different stimuli, physiological states, or age differences between the two samples might account for these differences. However, pending the results of a larger planned study; the data from this pilot study do not support the hypothesis that patients with anorexia nervosa have a wide-ranging hedonic deficit as posited by Friederich et al. [2]

The small number of subjects and cross-sectional design of this pilot study is its limitations. In addition, the hedonically positive response to ED-related stimuli in AN may also be secondary to heightened attention to ED-related stimuli as it relates to lower startle amplitude in response to ED-related categories.

## Conclusions

AN patients experience a high rate of relapse and mortality. The results of our study and those of some previous studies indicate that increased anxiety and excessive cognitive control combined with impaired reward sensitivity in AN patients lead to excessive food restriction. Inhibitory cognitive control maintains food restrictive behaviors in AN, and it is also observed clinically. Treatments aimed at reducing anticipatory anxiety, modulating attention to negative ED-related stimuli and improving reward sensitivity may improve clinical outcomes in AN-R.
